# Apoptosis inhibitor of macrophage (AIM) expression in alveolar macrophages in COPD

**DOI:** 10.1186/1465-9921-14-30

**Published:** 2013-03-05

**Authors:** Jun Kojima, Jun Araya, Hiromichi Hara, Saburo Ito, Naoki Takasaka, Kenji Kobayashi, Satoko Fujii, Chikako Tsurushige, Takanori Numata, Takeo Ishikawa, Kenichiro Shimizu, Makoto Kawaishi, Keisuke Saito, Noriki Kamiya, Jun Hirano, Makoto Odaka, Toshiaki Morikawa, Hiroshi Hano, Satoko Arai, Toru Miyazaki, Yumi Kaneko, Katsutoshi Nakayama, Kazuyoshi Kuwano

**Affiliations:** 1 Department of Internal Medicine, Division of Respiratory Diseases, Jikei University School of Medicine, 3-25-8 Nishi-shimbashi, Minato-ku, Tokyo 105-8461, Japan; 2 Department of Surgery, Division of Chest Diseases, Jikei University School of Medicine, Tokyo, Japan; 3 Department of Pathology, Jikei University School of Medicine, Tokyo, Japan; 4 Laboratory of Molecular Biomedicine for Pathogenesis, Center for Disease Biology and Integrative Medicine, Faculty of Medicine, The University of Tokyo, Tokyo, Japan

**Keywords:** AIM, Alveolar macrophage, Apoptosis, COPD

## Abstract

**Background:**

Marked accumulation of alveolar macrophages (AM) conferred by apoptosis resistance has been implicated in pathogenesis of chronic obstructive pulmonary disease (COPD). Apoptosis inhibitor of macrophage (AIM), has been shown to be produced by mature tissue macrophages and AIM demonstrates anti-apoptotic property against multiple apoptosis-inducing stimuli. Accordingly, we attempt to determine if AIM is expressed in AM and whether AIM is involved in the regulation of apoptosis in the setting of cigarette smoke extract (CSE) exposure.

**Methods:**

Immunohistochemical evaluations of AIM were performed. Immunostaining was assessed by counting total and positively staining AM numbers in each case (n = 5 in control, n = 5 in non-COPD smoker, n = 5 in COPD). AM were isolated from bronchoalveolar lavage fluid (BALF). The changes of AIM expression levels in response to CSE exposure in AM were evaluated. Knock-down of anti-apoptotic Bcl-xL was mediated by siRNA transfection. U937 monocyte-macrophage cell line was used to explore the anti-apoptotic properties of AIM.

**Results:**

The numbers of AM and AIM-positive AM were significantly increased in COPD lungs. AIM expression was demonstrated at both mRNA and protein levels in isolated AM, which was enhanced in response to CSE exposure. AIM significantly increased Bcl-xL expression levels in AM and Bcl-xL was involved in a part of anti-apoptotic mechanisms of AIM in U937 cells in the setting of CSE exposure.

**Conclusions:**

These results suggest that AIM expression in association with cigarette smoking may be involved in accumulation of AM in COPD.

## Background

Chronic obstructive pulmonary disease (COPD) is one of the leading causes of death worldwide and chronic cigarette smoke is sufficient to trigger COPD development [1]. Alveolar macrophages (AM) orchestrate innate immune responses for host defense through pattern recognition receptors (PRRs), thus marked increase of AM in response to cigarette smoke exposure has been widely implicated in pathogenesis of COPD via excessive inflammatory cytokine, chemokine, and protease secretions, resulting in lung tissue destruction and airway remodeling [2-5]. In addition to an inverse correlation between the number of AM and airflow limitation in COPD [6], reduction of AM by neutralization of granulocyte/macrophage colony-stimulating factor (GM-CSF) attenuated cigarette smoke-induced lung inflammation in mouse models [7]. Therefore, to elucidate the mechanism of AM accumulation may offer potential clues to understand COPD pathogenesis. However it remains unclear whether the increase in number of AM in COPD lung results from increased flux from circulating monocytes, local AM proliferation, or prolonged AM survival conferred by apoptosis resistance [2,3].

Apoptosis, a type of programmed cell death, is a physiologic mechanism for cell deletion without inflammation, which is necessary for the maintenance of homeostatic plasticity in the lung [8]. However, the cell type specific imbalance of positive and negative regulation of apoptosis has been proposed to be a critical determination of disease progression in COPD. For instance, excessive apoptotic cell loss of the alveolar epithelial cells and endothelial cells has been postulated to be a potential cause for development of lung tissue destruction, emphysema [9]. Conversely, reduced apoptosis through a lack of pro-apoptotic p53 expression [10] and an increase in anti-apoptotic Bcl-xL and the cytoplasmic form of p21^CIP1/WAF1^ has been reported in AM from smokers in association with chronicity of inflammation in COPD pathogenesis [11]. However the AM specific mechanism of anti-apoptotic property for prolonged cell survival in COPD lung has not been clearly elucidated.

Apoptosis inhibitor of macrophage (AIM), a member of scavenger receptor cysteine-rich superfamily, has been shown to be exclusively produced by mature tissue macrophages [12]. In mouse models, AIM contributes to the development of atherosclerotic lesions by conferring apoptosis resistance to cytotoxic oxidized-low density lipoprotein (oxLDL) in macrophages [13]. AIM is also involved in apoptosis regulation for T cell and natural killer T (NKT) cells in the model of corynebacterium-induced granuloma formation [14]. Furthermore, the anti-apoptotic property of AIM has been demonstrated against multiple apoptosis-inducing stimuli, including dexamethasone, irradiation, and Fas/CD95 [12]. Hence, we speculate the potential involvement of AIM in prolonged survival for AM under stress conditions of cigarette smoke exposure, which can be a part of mechanism for accumulation of AM in pathogenic sequence for COPD development. However both AIM expression in human lung and involvement of AIM in regulation of apoptosis in the setting of cigarette smoke exposure remains to be determined.

In this context, we examined the expression of AIM in lung tissues from COPD patients by means of immunohistochemial evaluation. We also examined the expression of AIM in AM isolated from bronchoalveolar lavage fluid (BALF) and also evaluated the changes of expression levels of AIM in response to cigarette smoke extract (CSE) exposure. Anti-apoptotic properties of AIM in CSE-induced apoptosis were also explored in in vitro models.

## Methods

### Immunohistochemical examinations

Lung tissue samples for immunohistochemistry were collected from pneumonectomy and lobectomy specimens from resections performed for primary lung cancer and tissue cancer involvement was excluded by histological examinations. Informed consent was obtained from all surgical participants as part of an approved ongoing research protocol by the ethical committee of Jikei University School of Medicine. Immunohistochemistry was performed as previously described with minor modification on the paraffin-embedded lung tissues [15]. The anti-AIM antibody SA-1, available for both mouse and human AIM recognition, was produced by immunizing rabbits with recombinant mouse AIM [12]. AM number and AIM staining were assessed by counting total and positively staining cells in five randomly selected lung fields at a magnification of × 400 in each case. Counting was only performed for cells in the airspace, which were morphologically recognized as alveolar macrophages. (n = 5 in control, n = 5 in non-COPD smoker, n = 5 in COPD). Patient characteristic features are presented in Table 1.

**Table 1:** Patient characteristics

	**Non-smoker**	**Non-COPD smoker**	**COPD**	**p value**
	**(n = 5)**	**(n = 5)**	**(n = 5)**	
Age, years	62.6 ± 16.7	62.0 ± 12.1	63.6 ± 3.5	NS
Male, % of group	40	80	100	NA
SI	0	38.3 ± 21.8*	78.5 ± 68.7	*p < 0.05.
FEV1/FVC	79.0 ± 6.5	76.7 ± 2.9	57.9 ± 13.5 *	*p < 0.05.
% VC	109.1 ± 19.8	104.4 ± 16.55	97.3 ± 18.7	NS

Definition of abbreviations: COPD = chronic obstructive pulmonary disease, FEV1 = forced expiratory volume in 1 second, SI = Smoking Index (pack/ year), VC = vital capacity, NA = not assessed, NS = not statistically significant. Values are mean ± SD.

### Cell culture and reagents

AM were isolated from bronchoalveolar lavage fluid (BALF) obtained from non-COPD patients. Filtered BALF was centrifuged at 400 g for 10 min. The cells were allowed to adhere to culture dishes in RPMI1640 with 10% FCS at 37°C for 2 h and nonadherent cells were removed. The adherent cells were microscopically confirmed as AM (>90%) [16]. U937, purchased from American type culture collection, were grown in RPMI1640 with 10% fetal bovine serum and penicillin-streptomycin.

Antibodies used were goat anti-AIM (AnaSpec, San Jose, CA), rabbit anti-Bcl-2 (Cell signaling Technology, Tokyo, Japan), rabbit anti-Bcl-xL (Cell signaling Technology, Tokyo, Japan), rabbit anti-caspase-8 (Cell signaling Technology, Tokyo, Japan), rabbit anti-caspase-9 (Cell signaling Technology, Tokyo, Japan), and mouse anti-β-actin (Santa Cruz, Santa Cruz, CA).

### Preparation of cigarette smoke extract

Cigarette smoke extract (CSE) was prepared as previously described [17]. Forty milliliters of cigarette smoke was drawn into the syringe and slowly bubbled into sterile serum-free cell culture media in 15-ml BD falcon tube. One cigarette was used for the preparation of 10 milliliters of solution. CSE solution was filtered (0.22 μm) to remove insoluble particles and was designated as a 100% CSE solution.

### Western blotting

AM and U937 cells grown on 6-well culture plates were lysed in 1X SDS sample buffer or RIPA buffer (Thermo Fisher Scientific, Waltham, MA) with protease inhibitor cocktail (Roche Diagnostics, Tokyo, Japan) and 1 mM sodium orthovanadate (Sigma Aldrich, Tokyo, Japan). Western blotting was performed as previously described with minor modification [15]. After transfer to PVDF membrane (Immobilon-P, Millipore, MA), blotting with specific primary antibodies were performed overnight at 4°C. Proteins were detected by HRP-conjugated secondary antibody (Cell signaling Technology, Tokyo, Japan) followed by chemiluminescence detection (ECL; GE Healthcare,Tokyo, Japan) with a LAS-4000 UV mini system (Fujifilm, Shiga, Japan).

### Plasmids, siRNA, and transfection

The AIM expression vector was generated by inserting humanAIM cDNA into the pCAGGS vector cassette. The BcL-xL and negative control siRNAs were purchased (Applied Biosystems Life Technologies Japan, Tokyo, Japan) and transfections of AM and U937 were performed using the Neon® Transfection System (Invitrogen Life Technologies Japan, Tokyo, Japan), using matched optimized transfection kits.

### Preparation of conditioned medium containing AIM

Human Embryonic Kidney (HEK) 293 cells were transfected with control or AIM expression vector by using TransIT®-LT1 Transfection Reagent (Mirus Bio LLC, WI) according to the manufacturer’s instruction. Conditioned medium without FCS preparation was started 24 h post-transfection and was collected after 48 h incubation. Collected conditioned medium and fresh RPMI1640 without FCS were mixed equally for further experiments.

### RNA isolation, quantitative polymerase chain reaction

RNA isolation, reverse transcription and Real-Time PCR were performed using the SYBR green method as previously described [15]. The primers used were AIM sense primer, 5’- TTCTCCTTGATCCTTGCCATTTG -3’; AIM antisense primer, 5’- ACTGGCCTTTCTGTTCCACC -3’; β-actin sense primer 5’-CATGTACGTTGCTATCCAGGC-3’β-actin antisense primer 5’-CTCCTTAATGTCACGCACGAT-3’. These primer sets yielded PCR products of 121 bp and 250 bp for AIM and β-actin respectively. Primer sequences for β-actin were from Primer Bank (http://pga.mgh.harvard.edu/primerbank).

### Detection of apoptosis and immunofluorescence staining

Cell cycle analysis and flow cytometry were performed as previously described [18]. Briefly, treated cells were harvested and immediately immobilized by 70% ice-cold ethanol overnight. Then the cells were incubated with 100 μg/ml of RNase and 50 μg/ml of propidium iodide (PI) (Sigma Aldrich, Tokyo, Japan) in PBS-Triton X-100 (0.05%) for 40 min at 37°C. The quantity of cells with hypodiploid DNA was measured on a FACScan at the FL-2 channel (Becton Dickinson). The percentage of control cells with hypodiploid DNA was designated as 100 % cell death in each experiment.

DNA fragmentation analysis was performed as previously described [18]. After treatment, collected cells were lysed in 100 μl of cell lysis buffer (10 mM Tris•HCl, pH7.4, 10 mM EDTA, pH8.0, and 0.5% TritonX-100). After centrifugation, the supernatant was treated with 2 μl of RNase A (20 mg/ml) for 30 min at 37°C and then incubated with 2 μl of proteinase K (20 mg/ml) for 30 min at 37°C. DNA in the supernatant was precipitated overnight by the addition of 20 μl 5 M NaCl and 120 μl isopropanol. After centrifugation, the DNA pellet was dissolved in Tris-EDTA buffer followed by electrophoresis on a 2% agarose gel. The agarose gel was stained with ethidium bromide, and the resulting DNA fragmentation pattern was revealed by ultraviolet illumination.

Fluorescence microscopic detection of apoptotic cells was performed as previously described [18]. Harvested cells were stained with 2’-(4-Hydroxyphenyl)-5-(4-methyl-1-piperazinyl)-2,5’-bi-1H-benzimidazole (Hoechst 33258: Sigma Aldrich, Japan), and then seeded on a glass slide and photographed with a fluorescent microscope. Percentage of apoptotic cells was assessed by manual counting, three hundred cells per condition.

### Statistics

Data are shown as the average (±SEM) taken from at least three independent experiments. Student’s t-test was used for comparison of two data sets. Comparison between groups was made using one-way ANOVA followed by the Tukey test. Linear regression analysis was used to compare average cell number of AM in high power field (X400) to percentage of AIM positive AM. Significance was defined as p < 0.05. Statistical software was Prism v.5 (GraphPad Software, Inc., San Diego, CA).

## Results

### AM accumulation and AIM expression in COPD lung

**Figure 1:**
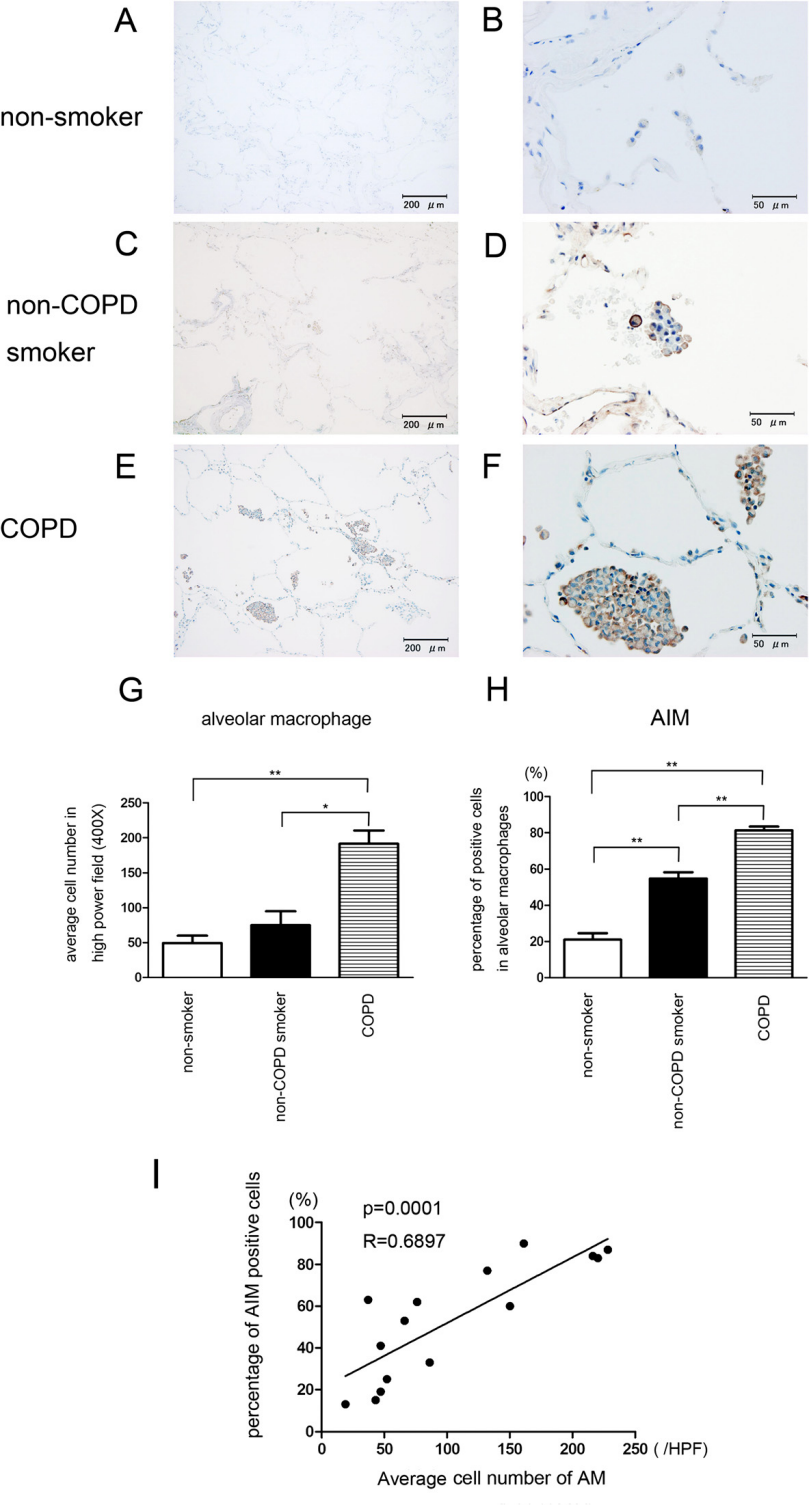
**AIM expression in alveolar macrophages in lung tissues. **Immunohistochemical staining of AIM in AM in lung tissues from non-smoker, non-COPD smoker, and COPD patients: Photomicrographs of non-smoker lung (**A** and **B**), non-COPD smoker lung (**C** and **D**), and COPD lung tissues (**E** and **F**). (**G**) Shown is the average total number of AM (±SEM) in five randomly selected lung fields in high power field (HPF) (X400) from five cases. Open bar is non-smoker, filled bar is non-COPD smoker, and horizontal crosshatched bar is COPD. *p < 0.05, **p < 0.001. (**H**) Shown is the average ± SEM of percentages of AIM positive cells in total cells in HPF (X400) from five cases. Open bar is non-smoker, filled bar is non-COPD smoker, and horizontal crosshatched bar is COPD. **p < 0.001. (**I**) Shown is the relationship between number of AM and percentages of AIM positive cells. Bar = 200 μm in **A**, **C**, and **E**. Bar = 50 μm in **B**, **D**, and **F**.

The number of AM was significantly increased in the lungs of COPD patients but not significant in non-COPD smoker compared to non-smoker (Figure 1A to G). AIM expression was clearly observed in AM in lung of COPD patients and the percentages of positively staining AM were significantly increased in both COPD patients and non-COPD smoker compared to non-smoker (Figure 1F, H). AIM staining was also lightly present in alveolar walls in lung tissues from smokers. Interestingly, the number of AM correlated significantly with increasing percentage of AIM positive AM, indicating the potential link between increasing AM accumulation and AIM expression (Figure 1I).

### CSE induces AIM expression in AM

**Figure 2:**
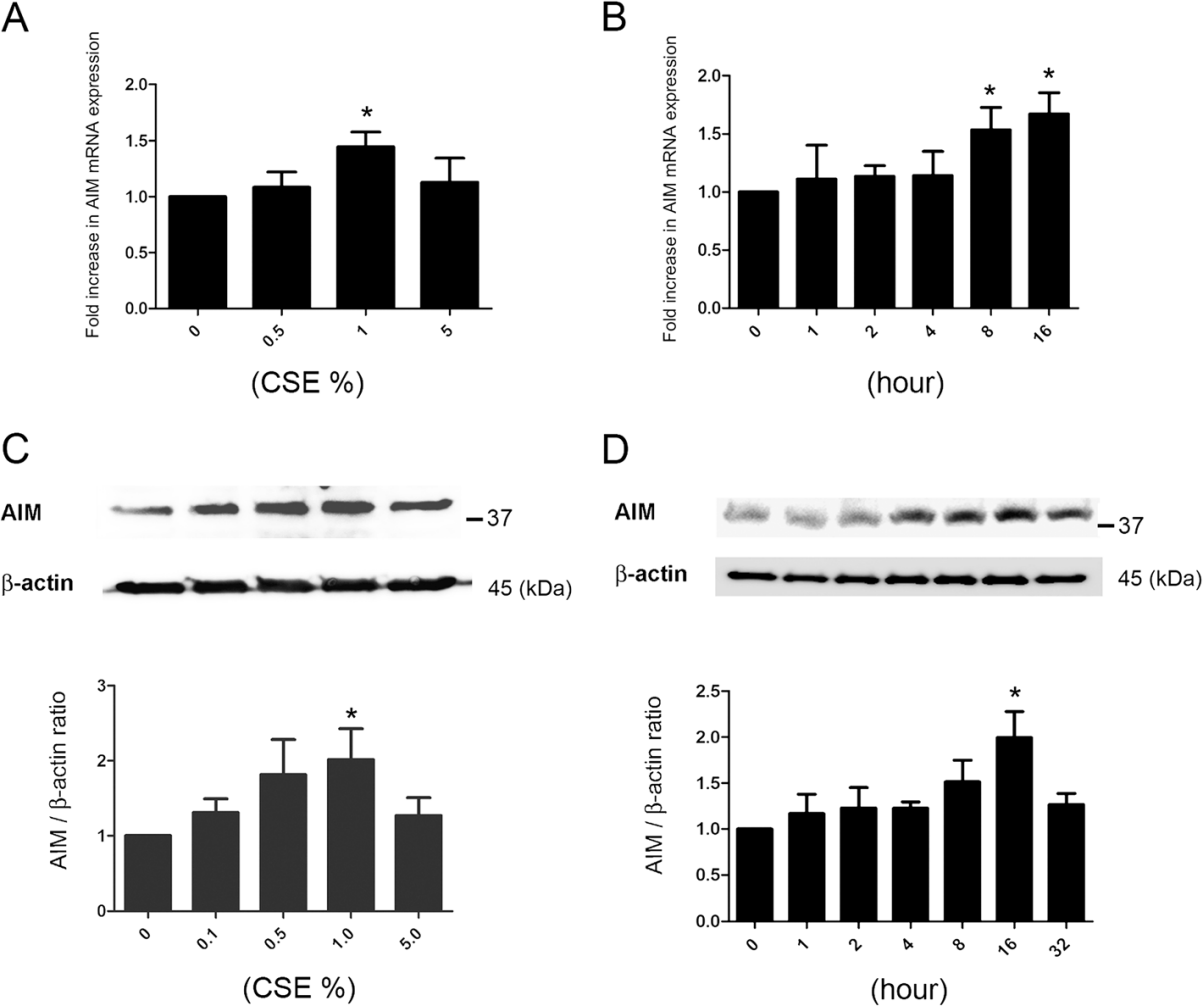
**AIM expression in alveolar macrophages isolated from bronchoalveolar lavage fluid. **(**A**) AM were treated with indicated concentrations of cigarette smoke extract (CSE) for 16 h (n = 5). Real time-PCR was performed using primers to AIM or β-actin, as a control. AIM expression was normalized to β-actin. Shown is the fold increase (±SEM) relative to control treated cells. *p < 0.05. (**B**) AM were treated with CSE (1.0%) and RNA were harvested at indicated time points (n = 4). Real time-PCR was performed using primers to AIM or β-actin. AIM expression was normalized to β-actin. Shown is the fold increase (±SEM) relative to control cells. *p < 0.05. (**C**) Western blotting (WB) using anti-AIM and anti-β-actin of cell lysates from indicated concentrations of CSE treated AM (upper panel). Cell lysates were collected after 16 h treatment. Shown is a representative experiment of 3 showing similar results. AIM was normalized to β-actin. The lower panel is the average (±SEM) of relative changes to control cells. (**D**) WB using anti-AIM and anti-β-actin of cell lysates from CSE (1.0%) treated AM (upper panel). Cell lysates were collected at indicated time points. Shown is a representative experiment of 3 showing similar results. AIM was normalized to β-actin. The lower panel is the average (±SEM) of relative changes to control cells.

We next examined the expression of AIM in AM isolated from BALF. AIM expression was demonstrated in AM and CSE significantly increased AIM expression at both mRNA and protein levels in AM and maximum effect was observed with 1% CSE (Figure 2A, C). However, significant induction was not observed with 5% CSE, which might be attributed to cytotoxic effect of higher concentration of CSE. CSE (1%) induced AIM expression peaked at 16h incubation at both mRNA and protein levels (Figure 2B, D). Protein level in cell lysate was decreased after 32 h incubation, which may reflect the secretory nature of AIM.

### AIM increases Bcl-xL expression levels in AM

**Figure 3:**
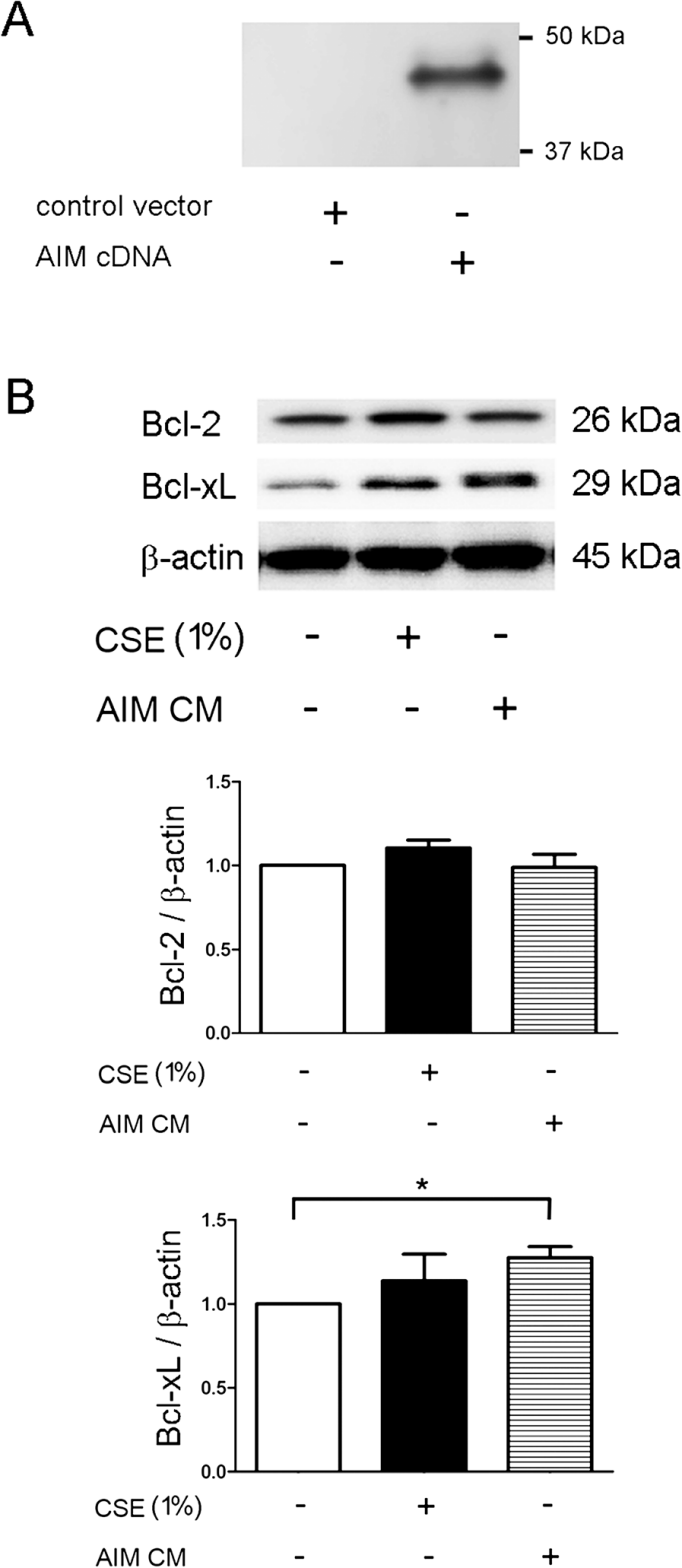
**The changes of Bcl-2 and Bcl-xL expression levels by AIM in alveolar macrophages. **(**A**) Western blotting (WB) using anti-AIM of conditioned medium from control empty vector (lane 1) or AIM expression vector (lane 2) transfected HEK293 cells. Conditioned medium was collected after 48 h incubation. (**B**) WB using anti-Bcl-2, anti-Bcl-xL, and anti-β-actin of cell lysates of alveolar macrophages (AM) treated with control conditioned medium (lane 1), control conditioned medium containing CSE (1.0%) (lane 2), and conditioned medium containing AIM (lane 3)(upper panel). Cell lysates of AM were collected after 24 h treatment. Shown is a representative experiment of 3 showing similar results. The middle panel is the average (±SEM) of relative changes in Bcl-2 normalized to β-actin. The lower panel is the average (±SEM) of relative changes in Bcl-xL normalized to β-actin. Open bar is control conditioned medium treated, filled bar is control conditioned medium treated in the presence of CSE (1.0%), and horizontal crosshatched bar is conditioned containing AIM treated. *p < 0.05.

Bcl-xL has been implicated in prolonged survival for AM in COPD lung [11]. Hence, to clarify the AIM-mediated anti-apoptotic mechanism in AM, anti-apoptotic Bcl-2 family proteins of Bcl-2 and Bcl-xL were evaluated. To prepare the conditioned medium containing recombinant AIM, HEK 293 cells were transfected with the AIM expression vector. Secretion of AIM in conditioned medium was confirmed by western blotting using anti-AIM antibody (Figure 3A). Intriguingly, we observed a significant increase in Bcl-xL expression levels in AM incubated with conditioned medium containing AIM (Figure 3B). However no significant increase was observed in the expression levels of Bcl-2, suggesting the specific induction of Bcl-xL by AIM in AM. Interestingly, non-significant increase of both Bcl-2 and Bcl-xL levels was also observed in response to CSE exposure.

### AIM inhibits CSE-induced apoptosis in U937cells

**Figure 4:**
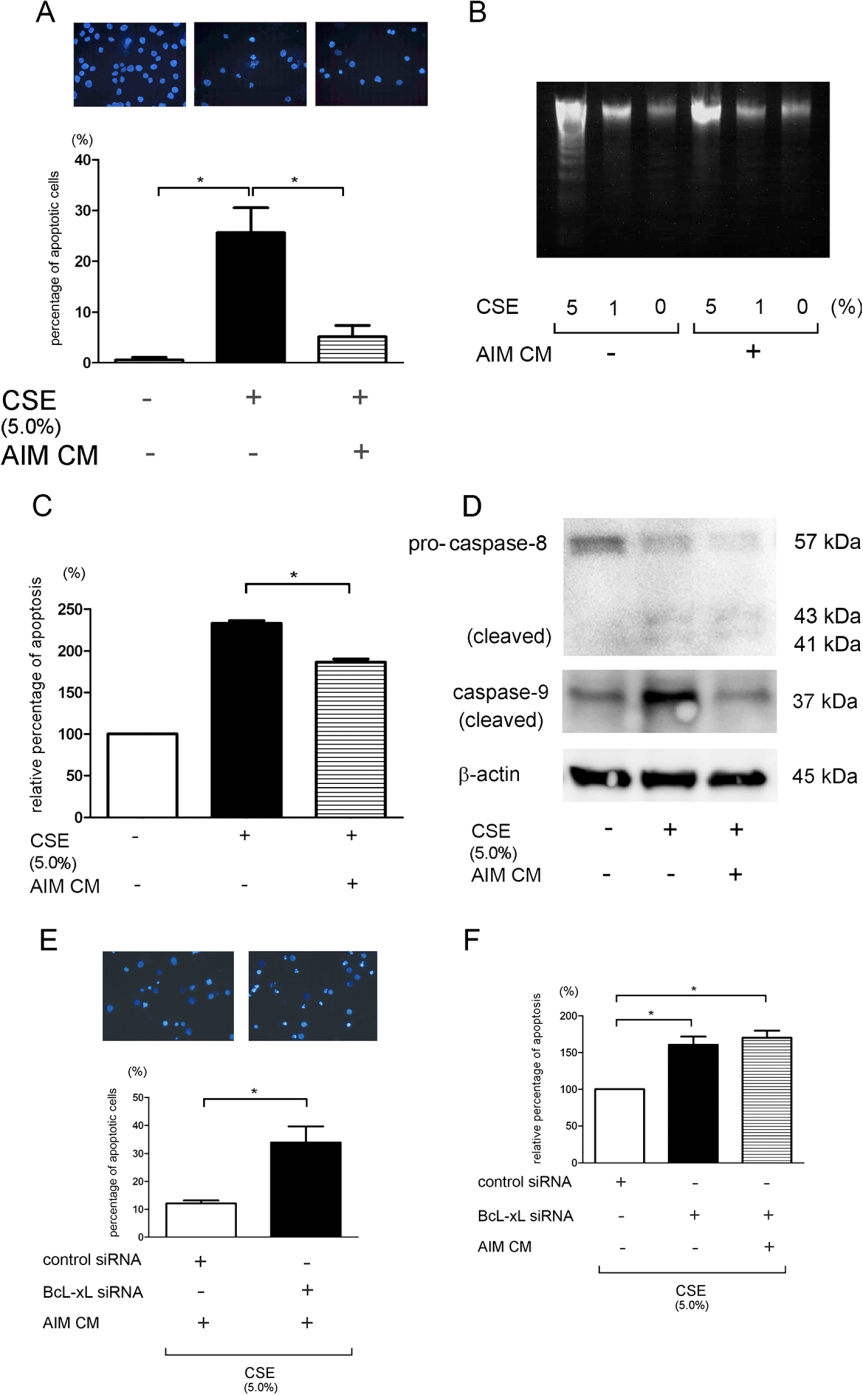
**Inhibitory role of AIM in cigarette smoke extract–induced apoptosis in U937 cells. **(**A**) Fluorescence microscopic detection of nuclear staining with Hoechst 33258: control (left panel), CSE (5.0%) treated (middle panel), and CSE (5.0%) treated in the presence of conditioned medium containing AIM (right panel). Lower panel shows the percentage (±SEM) of apoptotic cells from three independent experiments. *p < 0.05. (**B**) DNA fragmentation assay with or without CSE (1.0 or 5.0%) treatment in the absence or presence of conditioned medium containing AIM. (**C**) Measurement of DNA contents by flow cytometric analysis with propidium iodide (PI) staining. Shown is the relative percentage (±SEM) of hypodiploid apoptotic cells compared to control treated cells from four independent experiments. *p < 0.05. (**D**) Western blotting (WB) using anti-caspase-9, anti-caspase-8, and anti-β-actin in control treated (lane 1), CSE (5.0%) treated (lane 2), and CSE (5.0%) treated in the presence of conditioned medium containing AIM (lane 3). Protein samples were collected after 16 h treatment. Shown is a representative experiment of 3 showing similar results. (**E**) Fluorescence microscopic detection of nuclear staining with Hoechst 33258: control siRNA transfected (left panel) and BcL-xL siRNA transfected (right panel) cells were treated CSE (5.0%) in the presence of conditioned medium containing AIM. Lower panel shows the percentage (±SEM) of apoptotic cells from three independent experiments. Open bar is control siRNA transfected and filled bar is BcL-xL siRNA transfected. *p < 0.05. (**F**) Measurement of DNA contents by flow cytometric analysis with PI staining. Shown is the relative percentage (±SEM) of hypodiploid apoptotic cells compared to control treated cells from three independent experiments. Open bar is control siRNA transfected, filled bar is BcL-xL siRNA transfected, and horizontal crosshatched bar is BcL-xL siRNA transfected with conditioned medium containing AIM. *p < 0.05.

To evaluate the inhibitory role of AIM in CSE-induced apoptosis, we employed U937 monocyte-macrophage cell line without AIM expression (data not shown). CSE (5.0%) clearly induced apoptosis in U937 cells by means of nuclear staining with Hoechst 33258, DNA laddering, and FACS analysis for percentage of cells with hypodiploid DNA (Figure 4A to C).

CSE has been demonstrated to induce apoptosis through both the mitochondrial and death receptor pathways [19]. Therefore, to clarify which pathway is dominantly involved in CSE-induced apoptosis, we evaluated the caspase-8 and −9 activation by detecting cleaved active form by western blotting. CSE apparently activated both caspase-8 and caspase-9, indicating that both extrinsic and intrinsic apoptosis pathway are involved in CSE-induced apoptosis in U937 cell (Figure 4D). U937 cells cultured in the conditioned medium containing AIM were also treated with CSE. Interestingly, CSE-induced apoptosis was clearly suppressed by culturing in conditioned medium containing AIM. (Figure 4A to C). Interestingly, only caspase-9 activation was inhibited in the presence of AIM. To confirm the involvement of Bcl-xL in the AIM-mediated anti-apoptotic mechanism, U937 cells were transfected with Bcl-xL siRNA, resulting in decreased anti-apoptosis property of conditioned medium containing AIM (Figure 4E, F).

## Discussion

Although a previous paper failed to show AIM expression in the lung of murine models, which can be attributed to relatively small amount of expression levels with methodological limitations or species specific expression pattern of AIM in AM, the present study elucidated that human AM express AIM [12]. We consider that the increase in AIM expression in response to CSE exposure is an important clue for understanding the role of AIM in human lung pathophysiology. Our immunohistochemistry clearly demonstrated AIM expression in AM, which appeared to be associated with increase in number of AM in COPD lung. Intriguingly, slight AIM staining was also observed in alveolar walls in lung tissues from smokers, which can be attributed to the secretory nature of AIM, suggesting a potential role for AIM in paracrine regulation of alveolar epithelial cells and endothelial cells, which has yet to be determined. We also demonstrated the anti-apoptotic nature of AIM in CSE-induced apoptosis in U937 monocyte-macrophage cell line, which was at least partly attributed to increased expression of an anti-apoptotic Bcl-2-family protein, Bcl-xL.

The anti-apoptotic nature of AIM has been demonstrated to be against to multiple apoptosis-inducing stimuli, including dexamethasone, irradiation, and Fas/CD95, hence AIM may exert multiple mechanisms in anti-apoptotic regulation, but the detail remains to be determined [12]. Although the involvement of Bcl-2 and Bcl-x has been excluded from AIM-mediated anti-apoptosis in thymocytes, we demonstrated significantly increased Bcl-xL expression in AM in response to AIM (Figure 3). Because of the apoptosis resistance of AM in in vitro culture conditions, we used U937 cells as an experimental model to clarify the anti-apoptotic mechanism of AIM. Indeed, no apparent increase in cell death of AM by CSE (5.0%) exposure was observed by means of trypan blue dye exclusion (data not shown). Dominant inhibition of mitochondrial intrinsic apoptotic pathway of caspase-9 activation by AIM and inhibition of anti-apoptotic property of AIM by knockdown experiments, support the notion that Bcl-xL is involved in a part of anti-apoptotic mechanisms of AIM in the setting of CSE exposure. Therefore, we speculate that AIM may at least partly account for the previous finding of increased Bcl-xL expression in AM from smoker in association with apoptosis resistance in COPD lung [11]. However, we understand the potential limitations of using U937 to elucidate the anti-apoptotic mechanisms found in AM, hence more relevant in vitro and in vivo models are needed to further confirm the physiological importance of AIM in COPD pathology.

AM phenotype is largely divided into M1 and M2 polarization based on differences in patterns of cell surface receptor expression. The M1 polarization-induced by interferon-γ has leads to antigen presentation during cell-mediated immunity accompanied by production of T helper (Th) 1 type pro-inflammatory cytokines, IL-1β, IL-12 and tumor necrosis factor-α. In contrast, the M2 polarization-induced by the Th2 type cytokines IL-4 and IL-13 results in secretion of anti-inflammatory cytokines and expression of matrix metalloproteinase (MMP)-12 [20,21]. Although it is still uncertain which of these phenotypes is dominantly involved in COPD pathogenesis, there is compelling evidence that M1 polarized AM may play an important role in lung tissue destruction and impaired efferocytosis in response to cigarette smoke exposure [22,23]. Intriguingly, AIM has been demonstrated to be mainly expressed in M1 polarized macrophages in adipose tissue in obese mice [12]. Although the association between AM polarization and AIM expression in COPD is still unclear, it is plausible that the M1 polarized pro-inflammatory AM is mainly involved in AIM expression and chronicity of inflammation for COPD pathogenesis.

Although the detailed mechanism in in vitro CSE exposure remains to be determined, we speculate that oxLDL, a known strong inducer for AIM in atherosclerotic lesion, may be also involved in the mechanism for CSE-induced AIM expression in AM [13]. The presence of oxLDL in alveolar space of COPD lung has not been clearly demonstrated, however the experimental mouse model of lung edema induced by alpha-naphthylthiourea (ANTU) administration demonstrated existence of oxLDL in lung [24]. Indeed, oxLDL has been implicated in the pathogenesis for not only metabolic disorders but also several lung diseases such as asthma, acute lung injury, and cystic fibrosis through the surfactant regulation and inflammatory reactions [13,25]. Furthermore, oxLDL is one of the major and early lipid peroxidation products under the condition of increased oxidative stress, suggesting that cigarette smoke-induced highly oxidative microenvironment may enhance production of oxLDL in COPD lung. Among scavenger receptors, CD36 and macrophage scavenger receptor-1 (MSR1) are important for oxLDL uptake and AIM expression. One recent paper demonstrated that MSR1 expression is upregulated in AM of smokers [26]. In addition, polymorphisms in the MSR1 gene (*MSR1*) accompanied by increased MSR1 expression were associated with COPD susceptibility, lung function, and macrophage function [27]. Interestingly, MSR1 expression was also implicated in increase in cell number of monocyte-derived macrophage (MDM) in in vitro experiment [27]. Therefore, it is of particular interest to hypothesize that there is an association between MSR1 expression and up-regulation of AIM through the mechanisms of oxLDL uptake.

## Conclusions

**Figure 5:**
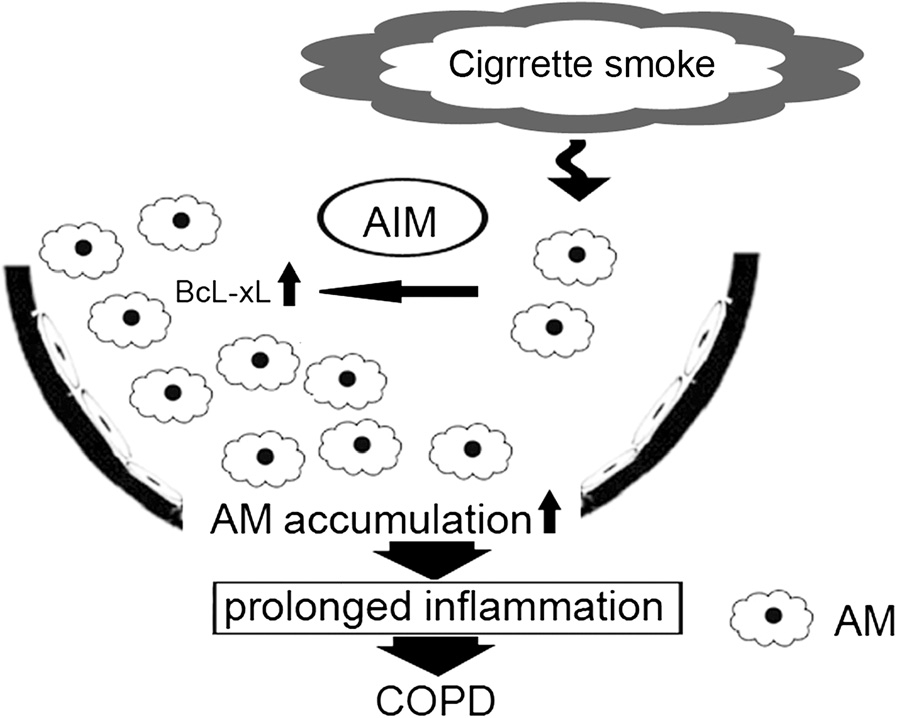
**Hypothetical model of involvement of AIM in COPD pathogenesis. **AIM expression AM in response to CSE exposure may enhance accumulation of AM as a pathogenic sequence for COPD development through prolonged inflammation. Definition of abbreviations: AIM = apoptosis inhibitor of macrophage, AM = alveolar macrophage, Bcl-xL = anti-apoptotic B cell lymphoma leukemia COPD = chronic obstructive pulmonary disease.

In summary, we have clarified the AIM expression in AM, which is enhanced in response to CSE exposure. The anti-apoptotic property of AIM to CSE-induced apoptosis may at least partly account for the mechanism of prolonged survival and increase in number of AM as a pathogenic sequence for COPD development (Figure 5). The anti-apoptotic property of AIM is against to multiple apoptosis-inducing stimuli and also for multiple cell types including lymphocytes, and AIM was originally identified as an important regulator for fat metabolism [28]. Hence, future studies need to address the involvement of AIM in different aspects, including systemic inflammation and metabolic disorders in association with COPD pathophysiology [29].

## Competing interests

None of the authors has a financial relationship with a commercial entity that has an interest in the subject of this manuscript.

## Authors’ contributions

JK, JA, HH, SI, NT, KK, SF, CT, TN, TI, KS, MK, KS, HH, SA, TM, YK, KN, and KK were involved in the conception and design of experiments, analyzed the data, and wrote the manuscript. JK, JA, HH, Sl, and NT performed the experiments. JK, SI, NT, KK, JH, MO, and TM obtained informed consent and collected human samples. All authors read and approved the final manuscript.
